# Changes in expression of interferon-stimulated genes and ubiquitin activating enzyme E1-like in ovine thymus during early pregnancy

**DOI:** 10.1590/1984-3143-AR2019-0134

**Published:** 2020-06-29

**Authors:** Leying Zhang, Zimo Zhao, Yujiao Wang, Ning Li, Nan Cao, Ling Yang

**Affiliations:** 1 Department of Animal Science, College of Life Sciences and Food Engineering, Hebei University of Engineering, Handan, China

**Keywords:** interferon-gamma-inducible protein 10, myxovirusresistance 1, sheep, signal transducer and activator of transcription 1, ubiquitin activating enzyme E1-like

## Abstract

As the main signal for the maternal recognition in ruminants, interferon-tau (IFNT) stimulates expression of interferon-stimulated genes (ISGs) in uterus and many extrauterine tissues. However, it is unclear that early pregnancy induces expression of signal transducer and activator of transcription 1 (STAT1), myxovirusresistance 1 (Mx1), interferon-gamma-inducible protein 10 (IP-10) and ubiquitin activating enzyme E1-like protein (UBE1L) in maternal thymus. In this study, ovine thymuses were sampled on day 16 of the estrous cycle and on days 13, 16 and 25 of gestation, and the expression of STAT1, Mx1, IP-10 and UBE1L was detected by real-time quantitative PCR, Western blot and immunohistochemistry. The results revealed that the expression of STAT1 and IP-10 reached peaks on day 16 of pregnancy, and expression of Mx1 was enhanced on day 25 of pregnancy, and STAT1 protein was located in the epithelial reticular cells, capillaries and thymic corpuscles. However, expression of UBE1L was declined during early pregnancy. In conclusion, early pregnancy influences expression of STAT1, Mx1, IP-10 and UBE1L in maternal thymus, which may participate in regulation of maternal immune tolerance during early pregnancy in sheep.

## Introduction

Interferon-tau (IFNT) is also known as trophoblastic protein-1, secreted by ruminant early conceptus (Godkin et al., 1984). IFNT acts on endometrium to suppress the luteolysis, and it is necessary for the action of progesterone (P4), which are implicated in immune protection of conceptus (Imakawa et al., 1987). IFNT stimulates endometria to express interferon-stimulated genes (ISGs), which are essential for conceptus survival during early pregnancy (Spencer, 2014). Many ISGs, including interferon-stimulated gene 15-kDa protein (ISG15) (Johnson et al., 1999), signal transducer and activator of transcription 1 (STAT1), STAT2, interferon regulatory factor 1 (IRF-1) and IRF-9 (Choi et al., 2001), myxovirusresistance (Mx) proteins (Ott et al., 1998) and 2’,5’-oligoadenylate synthetase (OAS-1) (Mirando et al., 1991) are upregulated in the ovine uterus during early pregnancy. Furthermore, during early pregnancy in sheep, IFNT secreted by the conceptus exerts effects on extrauterine tissues through an endocrine manner (Bott et al., 2010). Expression of ISG15 and OAS-1 are upregulated in corpus luteum (CL) and blood cells (Oliveira et al., 2008), and receptor transporter protein 4 improves in ovary and peripheral blood leukocytes (PBLs) (Gifford et al., 2008). ISG15 mRNA and protein are upregulated in the bone marrow (Yang et al., 2017), thymus (Zhang et al., 2018), spleen (Yang et al., 2018) and lymph node (Yang et al., 2019b) during early pregnancy in ewes.

As a primary lymphoid organ, the thymus is implicated in development of the immune system through regulating development and differentiation of thymocytes (Ribatti et al., 2006). There is a negative relation between P4 level and maternal thymus weight on the 4th to 7th days in pseudopregnant and pregnant mice (Chambers and Clarke, 1979), and sex steroid hormones are implicated in regulating thymic cell populations and maternal immune reactivities during pregnancy in mice (Shinomiya et al., 1991). Our previous studies find that 60-kDa P4 receptor isoform, the 62-kDa progesterone-induced blocking factor variant, cyclooxygenase 2 and aldo-keto reductase family 1, member B1, interferon-gamma, tumor necrosis factor beta, interleukin-5 (IL-5), IL-6 and IL-10 are upregulated in ovine thymus during early pregnancy (Zhang et al., 2018, 2019; Yang et al., 2019a).

IFNT exerts its effects via binding to common type I interferon receptors in endometrium and Madin-Darby bovine kidney cells in cattle (Li and Roberts, 1994), and regulates the antiluteolytic effects on the endometrium by activation of Janus kinase (JAK)- STAT pathway in the bovine (Binelli et al., 2001). It is via JAK-STAT pathway that over expression of long non-coding RNA 135528 in glioma cells results in upregulation of interferon-gamma-inducible protein 10 (IP-10) that is also known as C-X-C motif chemokine 10 (CXCL10) (Wang et al., 2018). ISGs play key roles in the antiviral response of the host, and are required for the innate immune system (Hubel et al., 2019). Ubiquitin activating enzyme E1-like protein (UBE1L) participates in ISG15 conjugation, and ISG15 and UBE1L mRNA increase in peripheral blood mononuclear cells (PBMCs) on day 18 of pregnancy in cows (Haq et al., 2016). UBE1L is involved in modulation of protein functions through covalent attachment of ubiquitin or ubiquitin-like proteins (Ebner et al., 2017). The purpose of this project was to analyze expression of STAT1, Mx1, IP-10 and UBE1L in ovine thymuses, which may be useful for making out the effects of early pregnancy on maternal thymus.

## Methods

### Animals and experimental design

Hebei University of Engineering Animal Care and Use Committee approved all animal procedures (AEEI-16015). The experimental design described previously (Zhang et al., 2018). Briefly, Small-tail Han ewes were randomly divided into four groups (n = 6 for each group). Ewes from three groups were exposed to a fertile ram and mated twice, and the ewes from the rest group were not. Thymus samples were obtained from the ewes on days 13, 16 and 25 after mated, and nonpregnant ewes (day 16 after estrus) at slaughter. Cross thymic sections (approximately 0.5 cm^3^) including the cortex and the medulla were fixed by immersion in 4% (w/v) paraformaldehyde for 24 h, and then embedded in paraffin. The remaining portions were frozen at -195.8 °C for detection of target mRNA and proteins.

### RNA extraction and real-time quantitative PCR (RT-qPCR) assay

Total RNA isolation, cDNA synthesis and real-time quantitative PCR (RT-qPCR) assay were performed as previously described (Zhang et al., 2018) with TRIzol reagent, a FastQuant RT kit and a SuperReal PreMix Plus kit (Tiangen Biotech Co., Ltd., Beijing). The primer sequences for STAT1, Mx1, IP-10, UBE1L and glyceraldehyde-3-phosphate dehydrogenase (GAPDH) were designed and synthesized by Shanghai Sangon Biotech Co., Ltd. (Table 1). PCR was carried out under the conditions of 40 cycles of 95 °C for 10 sec, 57-65 °C (57 °C for IP-10, 62 °C for UBE1L, 63 °C for STAT1, 65 °C for Mx1) for 20 sec, and 72 °C for 25 sec. The relative expression values of the genes were calculated by the 2^-ΔΔCt^ analysis method with GAPDH as the endogenous control (Wong and Medrano, 2005). The relative expression value for the group of day 16 of the estrous cycle was used as normalization.

**Table 1:** Primers used for RT-qPCR.

**Gene**	**Primer**	**Sequence**	**Size (bp)**	**Accession numbers**
STAT1	Forward	GTGGCGGAGAGTCTGCAGCA	190	NM_001166203.1
	Reverse	GGTGAGTTGGCATGCAGGGC		
Mx1	Forward	CCACCACCGACAGCTCCCCT	147	NM_001009753.1
	Reverse	GCAGGTGTGGGCGTGAAGCA		
IP-10	Forward	TCTAGGAACACACGCTGCAC	108	NM_001009191.1
	Reverse	GACACGTGGGCAGGATTGAC		
UBE1L	Forward	TGCGGTACATTCCTGCCACAAC	141	XM_027957619.1
	Reverse	TCTGCGACTTAACCAAGCCTTCTG		
GAPDH	Forward	GGGTCATCATCTCTGCACCT	176	NM_001190390.1
	Reverse	GGTCATAAGTCCCTCCACGA		

Note: bp = base pair.

### Western blot analysis

Proteins in the thymic samples were extracted using RIPA lysis buffer (Biosharp, BL504A), and protein concentration was measured with a BCA Protein Assay kit (Tiangen Biotech). Total proteins (10 μg/lane) were separated with sodium dodecyl sulphate-polyacrylamide gel electrophoresis, and transferred to polyvinylidene fluoride membranes. The membrane was blocked in 5% skimmed milk powder, and then incubated with a goat anti-STAT1 polyclonal antibody (Abcam, Cambridge, UK, ab230428, 1:1000), a mouse anti-Mx1 monoclonal antibody (Santa Cruz Biotechnology, Santa Cruz, CA, USA, sc-166412, 1:1000), a mouse anti-IP-10 monoclonal antibody (Santa Cruz Biotechnology, sc-374092, 1:1000), and a mouse anti-UBE1L monoclonal antibody (Santa Cruz Biotechnology, sc-390097, 1:1000), respectively. Membranes were washed three times, and incubated with goat anti-mouse IgG-HRP (Biosharp, BL001A) or rabbit anti-goat IgG-HRP (Biosharp, BL004A) in a 1:2000 dilution. After washed three times, proteins detected by a pro-light HRP chemiluminescence kit (Tiangen Biotech) according to the manufacturer’s instructions. A GAPDH antibody (Santa Cruz Biotechnology, Inc., sc-20357, 1:1000) was used to monitor sample loading. The blots were quantified using Quantity One software (v450; Bio-Rad Laboratories, Inc., Hercules, CA).

### Immunohistochemistry analysis

Some sections were stained with hematoxylin and eosin (HE). Others were quenched endogenous peroxidase activity, and reduced nonspecific binding, and then the sections were incubated with the anti-STAT1 antibody (ab230428, dilution 1:200). A rabbit anti-goat IgG-HRP (Biosharp, BL004A, dilution 1:500) was used to conjugate the STAT1 antibody, and a DAB kit (Tiangen Biotech) was used to detect the signals. For negative control, the STAT1 antibody was replaced with goat IgG at equivalent concentration, and nuclear staining was performed with hematoxylin. The sections were observed with a light microscope (Nikon Eclipse E800, Japan), and photographed with a digital camera DP12. Finally, the images were examined independently by 4 observers, and the immunostaining intensity of the different thymic samples from different ewes (n = 6 for each group) was rated in a blinded fashion. STAT1 staining intensity and pattern were analyzed by assigning an immunoreactive intensity of a scale of 0 to 3, as described previously (Zhang et al., 2019). An intensity of 3+ was given to the cells with the highest staining intensity, and an intensity of 0 was assigned to the cells with no immunoreactivity.

### Statistical analysis

Data for relative expression values of STAT1, Mx1, IP-10 and UBE1L mRNA and proteins were analyzed with MIXED procedure in SAS (Version 9.1; SAS Institute, Cary, NC). Duncan method was used to compare the relative expression levels of the different groups, and controlling the experimentwise type ± error equal to 0.05. Data are presented as least squares means. *P* < 0.05 was considered significantly different.

## Results

### Expression of STAT1, Mx1, IP-10 and UBE1L genes in the thymuses

**Figure 1:**
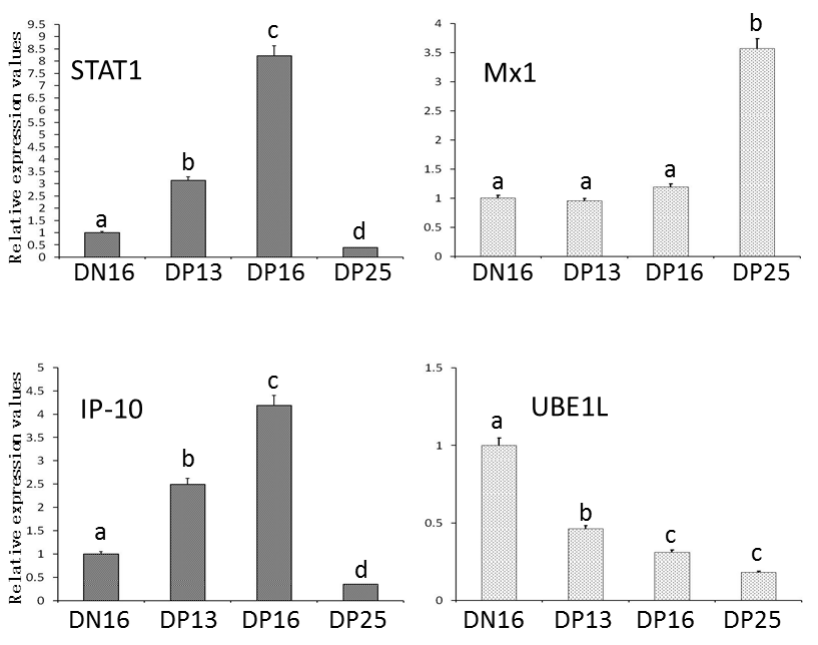
Relative expression values of STAT1, Mx1, IP-10 and UBE1L mRNA in the thymuses (n = 6 for each group). Note: DN16: day 16 of nonpregnancy; DP13: day 13 of pregnancy; DP16: day 16 of pregnancy; DP25: day 25 of pregnancy. The different letter above the same color column indicates significant difference (*P* < 0.05).

It is revealed in Figure 1 that expression of STAT1 and IP-10 genes reached peaks on day 16 of pregnancy, but expression of UBE1L gene was lower in the thymus from the pregnant ewes comparing with that from the nonpregnant ewes (*P* < 0.05). The relative value of Mx1 gene was higher in the thymus from day 25 of pregnant ewes than that from the ewes on day 16 of the estrous cycle, days 13 and 16 pregnancy (*P* < 0.05).

### Expression of STAT1, Mx1, IP-10 and UBE1L proteins in the thymuses

**Figure 2:**
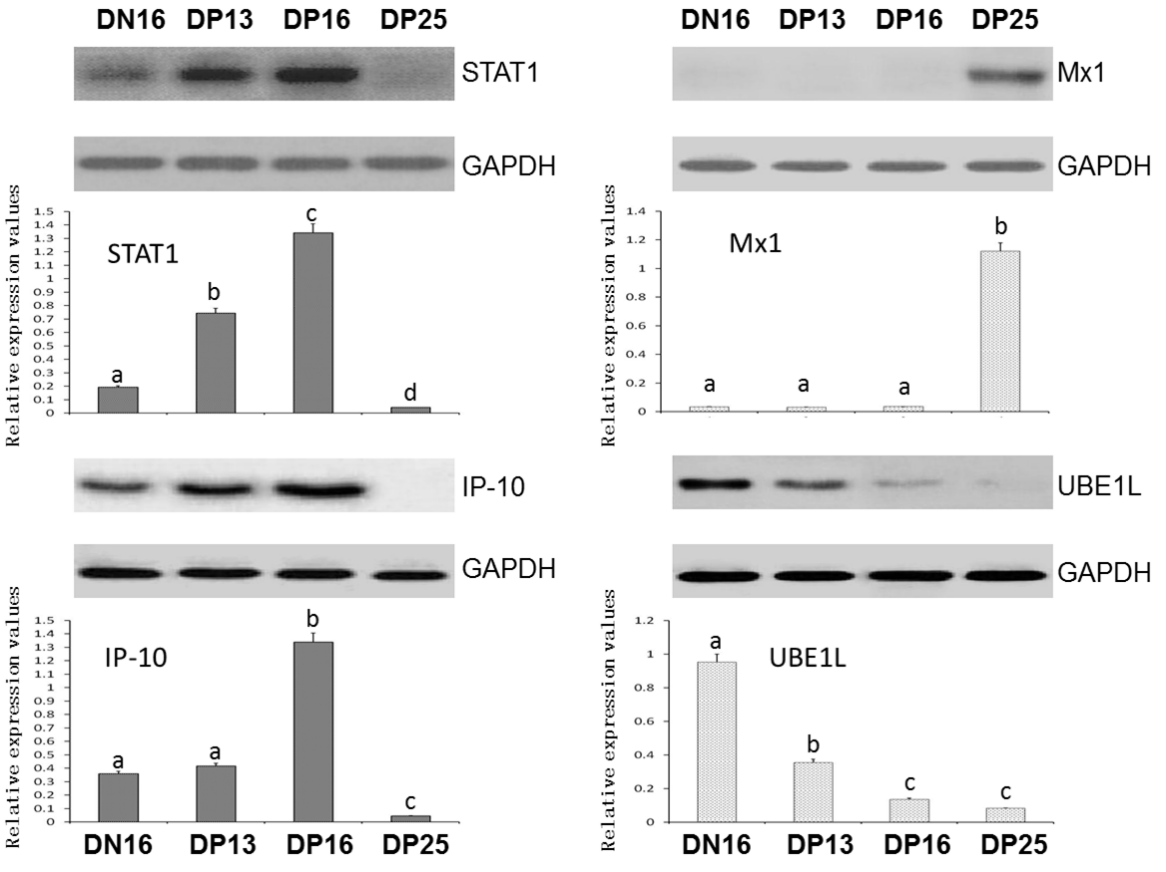
Expression of STAT1, Mx1, IP-10 and UBE1L proteins in ovine thymuses (n = 6 for each group). Note: DN16: day 16 of nonpregnancy; DP13: day 13 of pregnancy; DP16: day 16 of pregnancy; DP25: day 25 of pregnancy. The different letter above the same color column indicates significant difference (*P* < 0.05).

Western blot analysis found that expression of STAT1 and IP-10 proteins was upregulated on day 16 of pregnancy, and there was almost no expression of STAT1 and IP-10 proteins on day 25 of pregnancy (Figure 2). Expression of UBE1L protein was higher in the thymus from the nonpregnant animals than that from pregnant animals (*P* < 0.05). However, Mx1 protein was only expressed in the thymus on day 25 of pregnancy (*P* < 0.05), and not expressed in the thymus on day 16 of the estrous cycle, and days 13 and 16 pregnancy.

### Immunohistochemistry for STAT1 protein in the thymuses

**Figure 3:**
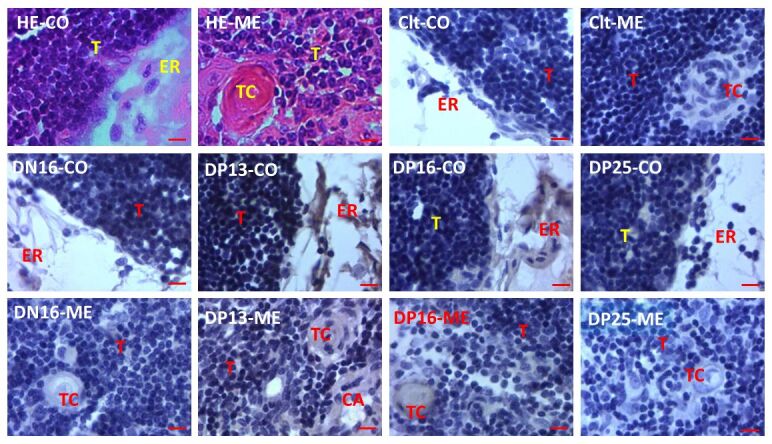
Representative immunohistochemical localization of STAT1 protein in the ovine thymuses (n = 6 for each group). The thymus is divided into the cortex (CO) and the medulla (ME). Note: HE: stained by hematoxylin and eosin; Clt: negative control; DN16: day 16 of nonpregnancy; DP13: day 13 of pregnancy; DP16: day 16 of pregnancy; DP25: day 25 of pregnancy; T: thymocyte; ER: epithelial reticular cell; CA: capillary; TC: thymic corpuscle. Bar = 10 µm.

Immunohistochemistry analysis revealed that STAT1 protein was located in the epithelial reticular cells, capillaries and thymic corpuscles (Figure 3), and the staining intensity for STAT1 was stronger in the thymuses on days 13 and 16 of pregnancy comparing to that on day 16 of the estrous cycle, and day 25 of pregnancy.

## Discussion

Interferon signal is implicated in the T cell maturation through upregulation of interferon-α receptor and STAT1 in thymus (Xing et al., 2016). In this study, STAT1 mRNA and protein were upregulated in ovine thymus on day 16 of pregnancy. Pregnancy enhances STAT1 expression and protein phosphorylation in the endometrium during the peri-implantation period, and IFNT also does it in endometrial cells *in vitro* (Vitorino Carvalho et al., 2016). Bovine IFNT stimulates STAT1 binding to DNA, which results in tyrosine phosphorylation, nuclear translocation via JAK-STAT to regulate the antiluteolytic action in bovine endometrial epithelial cells (Binelli et al., 2001). Recombinant ovine IFNT enhances expression of STAT1 in luteal slices and luteal endothelial cells, and promotes luteal cell survival to extend luteal life span in the bovine (Basavaraja et al., 2017). IFNT acts systemically to stimulate the expression of ISGs, including STAT1, and affects development of the conceptus and uterine glands, secretion of progesterone by CL (Bazer and Thatcher, 2017). JAK-STAT pathway is implicated in orchestrating of immune system through regulation of T helper cell subsets (Seif et al., 2017). Therefore, the upregulation of STAT1 in maternal thymus may participate in regulating maternal innate and adaptive immunities during early pregnancy in sheep.

Our results showed that Mx1 mRNA and protein upregulated in the maternal thymus on day 25 of pregnancy. Mx1 is a guanosine 5’ triphosphatases, and plays critical roles in innate immunity (Gao et al., 2011). Mx1 is a family of ISGs, and expressed in the endometrium and placenta during early to mid pregnancy, which is helpful for the mother and fetus in cattle (Shirozu et al., 2016). Mx1 gene is upregulated in ovine CL during pregnancy via microarray analysis, and induced by IFNT in culturing luteal cells (Romero et al., 2013). Mx1 gene is expressed in maternal liver during late periimplantation period, which is induced by embryonic signal IFNT in the bovine (Meyerholz et al., 2016). Mx1 mRNA is upregulated in the cervix, vagina and blood neutrophils during early pregnancy in cows (Kunii et al., 2018; Sheikh et al., 2018). Mx1 interacts with tubulin beta in the ovine glandular epithelial cells during interphase and mitosis, and is implicated in intracellular trafficking and secretion in ovine uterus during early pregnancy (Racicot et al., 2012). Mx1 is related to exosomes and protected from proteases, which is involved in secretion of epithelial cells and regulation of uterine function in sheep (Racicot and Ott, 2011). Therefore, upregulation of Mx1 in maternal thymus may be involved in regulation of thymic function during early pregnancy.

Our results revealed that IP-10 mRNA and protein were increased in ovine thymus on day 16 of pregnancy, and declined on day 25 of pregnancy. IP-10 is a chemotactic CXC chemokine, secreted by immune cells, and participates in regulation of T helper cells, B cells and macrophages activities (Romagnani and Crescioli, 2012). As a proinflammatory cytokine, IP-10 mRNA and protein are expressed in thymic stromal cells, and implicated in T cell development (Gattass et al., 1994). IFNT stimulates expression of IP-10 mRNA in the endometrium, and IP-10 participates in conceptus migration to endometrial epithelium in goats (Nagaoka et al., 2003). IP-10 mRNA is increased in the uterus during early pregnancy, which is implicated in regulating the ability of conceptus adhesion to the endometrium in sheep (Imakawa et al., 2006). Early pregnancy induces expression of IP-10 mRNA in PBLs, and IFNT treatment also does it in *in vitro* cultured PBLs, suggesting that upregulation of IP-10 is a pregnancy-dependent event in cows (Sakumoto et al., 2018). There is an upregulation of IP-10 gene in PBMCs during early pregnancy, which is related with early pregnancy recognition in sheep (Mauffré et al., 2016). Therefore, the upregulation of IP-10 may participate in the regulation of maternal thymic functions, which is related with early pregnancy in sheep.

In this study, expression of UBE1L mRNA and protein were decreased in the thymus during early pregnancy. UBE1L is a core E1 enzyme for ISG15 conjugation (ISGylation) that ISG15 conjugation to a target protein is not for target protein degradation, but involved in regulating the function of the target protein (Durfee and Huibregtse, 2010). Expression of UBE1L protein is increased in the uterus during early pregnancy in cows (Rempel et al., 2005), and early pregnancy induces upregulation of UBE1L genes in bovine PBMCs (Haq et al., 2016). ISGylation participates in the innate immunity, but fertile ability and antiviral responses is normal in a mouse model deficient in UBE1L, suggesting that UBE1L and protein ISGylation are not necessary for type 1 interferon signaling (Kim et al., 2006). UBE1L and ISGylation have tumor suppressor function, but protein ISGylation and tumor progression are not altered in the UBE1L-deficient mice with the K-ras^LA2^ lung cancer (Yin et al., 2009). Therefore, the downregulation of UBE1L in the thymus is not related with IFNT, which need to further study.

Thymus is made up of the cortical and medullary compartments, and plays key roles in the peripheral immune system (Lynch et al., 2009). Our immunohistochemistry results revealed that STAT1 protein was located in the epithelial reticular cells, capillaries and thymic corpuscles, and the staining intensity for STAT1 was stronger on days 13 and 16 of pregnancy. Steroid hormone is involved in thymus deterioration through an endocrine manner, which leads to immune system deterioration with age, suggesting that changes in steroid hormone during pregnancy affect the thymus function and peripheral immune responses (Calder et al., 2011). Tolerance induction occurs in thymus through clonal elimination of V beta 17a in animals (Kappler et al., 1987), and there exists maternal immune tolerance that the fetus and placenta are protected from rejection by the maternal immune system during pregnancy (Williams, 2012). Therefore, the upregulation of STAT1 in the epithelial reticular cells, capillaries and thymic corpuscles may participate in regulation of maternal immune tolerance.

## Conclusion

Early pregnancy induced upregulation of STAT1, Mx1 and IP-10 in the thymus, but expression of UBE1L was declined. In addition, STAT1 protein was located in the epithelial reticular cells, capillaries and thymic corpuscles. Therefore, early pregnancy influences expression of STAT1, Mx1, IP-10 and UBE1L in maternal thymus, which may participate in regulation of maternal immune tolerance during early pregnancy in sheep.
